# Anatomical and Technical Considerations of the Hi-PAC (Hi-Volume Proximal Adductor Canal) Block: A Novel Motor-Sparing Regional Analgesia Technique for Below-Knee Surgeries

**DOI:** 10.7759/cureus.21953

**Published:** 2022-02-06

**Authors:** Kartik Sonawane, Hrudini Dixit, Tuhin Mistry, Palanichamy Gurumoorthi, Jagannathan Balavenkatasubramanian

**Affiliations:** 1 Anesthesiology, Ganga Medical Centre and Hospitals Pvt Ltd, Coimbatore, IND; 2 Anesthesiology and Perioperative Medicine, Ganga Medical Centre and Hospitals Pvt Ltd, Coimbatore, IND

**Keywords:** subsartorial blocks, procedure-specific ra technique, ankle surgery, proximal tibia surgery, below-knee surgeries, motor-sparing ra technique

## Abstract

Below-knee surgeries are among the most commonly performed orthopedic or plastic and reconstructive procedures. They are associated with significant postoperative pain despite the use of systemic analgesics. The regional analgesia (RA) technique has been proven beneficial for better patient outcomes when used as an adjunct to multimodal analgesia in the early postoperative period. However, apprehension of an acute compartment syndrome (ACS) can limit the administration of appropriate RA techniques in such surgeries, leading to more opioid consumption to meet the increasing analgesic demands. Many modifications in the RA related to techniques and the local anesthetic type, concentration, and volume have been described to tackle such situations. The ideal RA technique should provide procedure-specific analgesia below the knee joint without affecting motor power and/or causing any delay in diagnosing or treating ACS.

The high-volume proximal adductor canal (Hi-PAC) block is a novel RA technique described as motor-sparing and procedure-specific for the below-knee surgeries. The Hi-PAC block, a single-injection technique, is administered in the proximal adductor canal targeting the saphenous nerve and depositing local anesthetics (LA) adjacent to the femoral artery below the vasoadductor membrane (VAM). By directly blocking the saphenous nerve and indirectly the sciatic nerve, it covers the entire innervation of the pain-generating components involved in the below-knee surgeries.

This article describes the anatomical and technical considerations of the Hi-PAC block and provides background knowledge of the relevant anatomy and sonoanatomy for a better understanding of its intricacies.

## Introduction

The pain management in the lower extremity surgeries is always challenging due to variable severity of the pain depending on the surgical dissection, suboptimal analgesia with only systemic analgesics, and restricted use of regional analgesia (RA) techniques due to concerns about the possibility of developing acute compartment syndrome (ACS). Several RA techniques have been described to provide analgesia for below-knee surgeries, including continuous epidural infusion, lumbosacral plexus block, continuous perineural catheter techniques, and combinations of peripheral nerve blocks. The ideal RA technique for providing postoperative analgesia for below-knee surgery should cover all essential innervations of the pain generators involved in each surgical step without compromising the motor strength to encourage early mobilization and discharge. Most importantly, it should not cause any delay in detection or management of the ACS by masking the clinical symptoms.

The high-volume proximal adductor canal (Hi-PAC) block is a novel RA technique for below-knee surgeries, described as an anterior and indirect approach to popliteal sciatic nerve block [1,2]. The selective sensory coverage of all the innervations of the pain-generating components makes the Hi-PAC block a motor-sparing, opioid-sparing, and procedure-specific RA technique for below-knee surgeries. The motor-sparing effect of the Hi-PAC block depends on the type, concentration, volume, and distribution of the local anesthetics (LA). Selective sensory blockade using low-concentrated (diluted) LA prevents masking of the symptoms of the impending compartment syndrome, risk of falls, delayed mobility, and prolonged hospital stay.

This article focuses on the innervation below the knee joint and the complex neurovascular inter-relationships in various parts of the adductor canal. Such thorough background knowledge is essential to understand the anatomical and technical aspects of the Hi-PAC block and its novelty in providing complete analgesia below the knee joint.

## Technical report

Understanding the technical aspect of the Hi-PAC block relies on a comprehensive knowledge of the relevant anatomy, sonoanatomy, and drug distribution patterns to include target innervations.

Relevant anatomy

The anatomy related to the Hi-PAC block mainly revolves around the innervation below the knee joint and identification of the demarcation point between the two prominent landmarks over the anterior thigh (femoral triangle and the adductor canal) to reach the target site.

**Figure 1:**
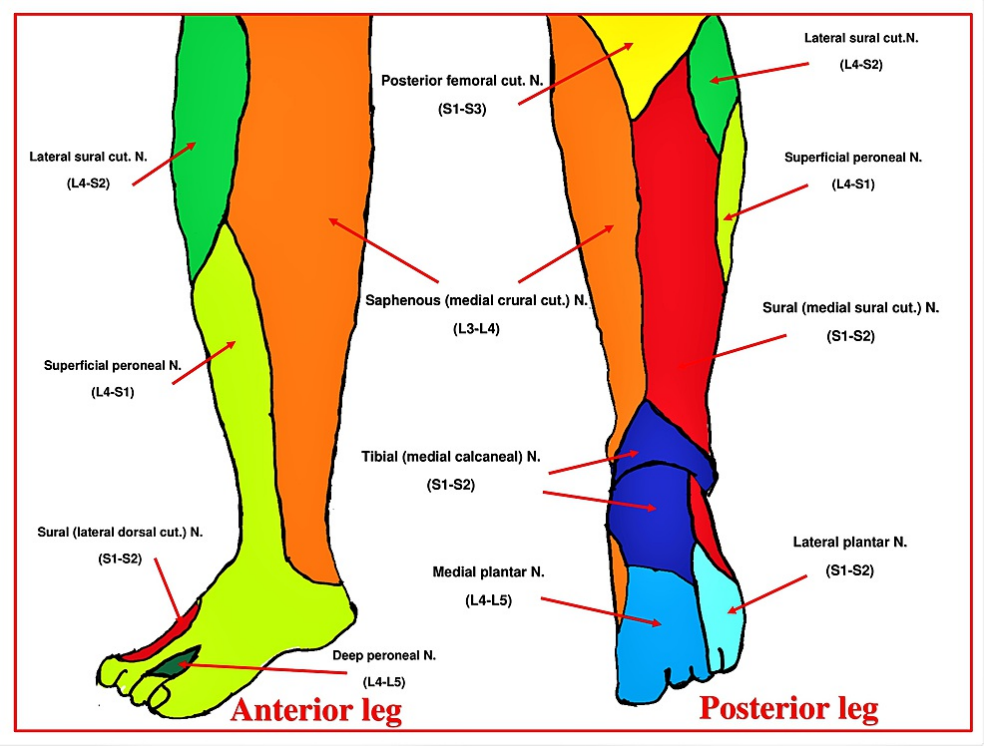
Dermatomal innervation of the leg below the knee joint N: Nerve. Source: This figure was created by the first author KS.

**Figure 2:**
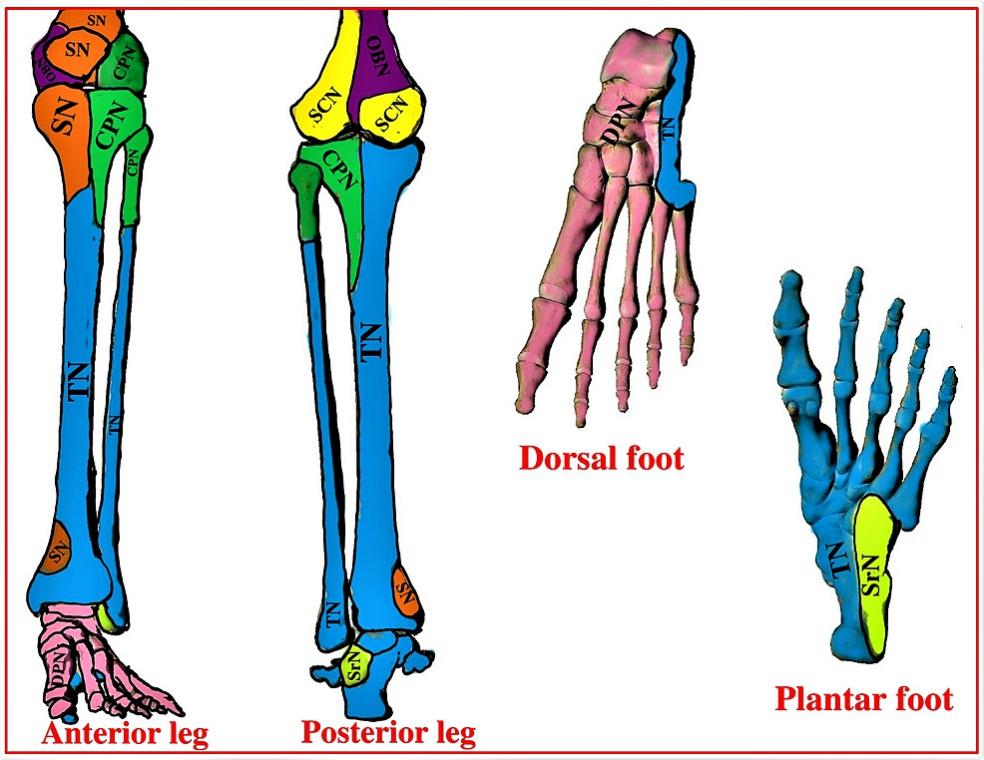
Osteotomal innervation of the leg below the knee joint SN: Saphenous nerve, TN: Tibial nerve, CPN: Common peroneal nerve, DPN: Deep peroneal nerve, SCN: Sciatic nerve, OBN: Obturator nerve, SrN: Sural nerve. Source: This figure was created by the first author KS.

The innervation of the leg below the knee joint encompasses the major contribution of the sciatic nerve (SCN) components (tibial and common peroneal) and the minor contribution of the saphenous nerve (SN). The superficial and deep branches of these nerves supply skin (dermatome) (Figure 1), muscles (myotome), and bones (osteotome) (Figure 2). Being a motor-sparing RA technique, the Hi-PAC block mainly focuses on the sensory innervation of postoperative pain generators, including skin over the incision site and the bone at the surgery site.

**Figure 3:**
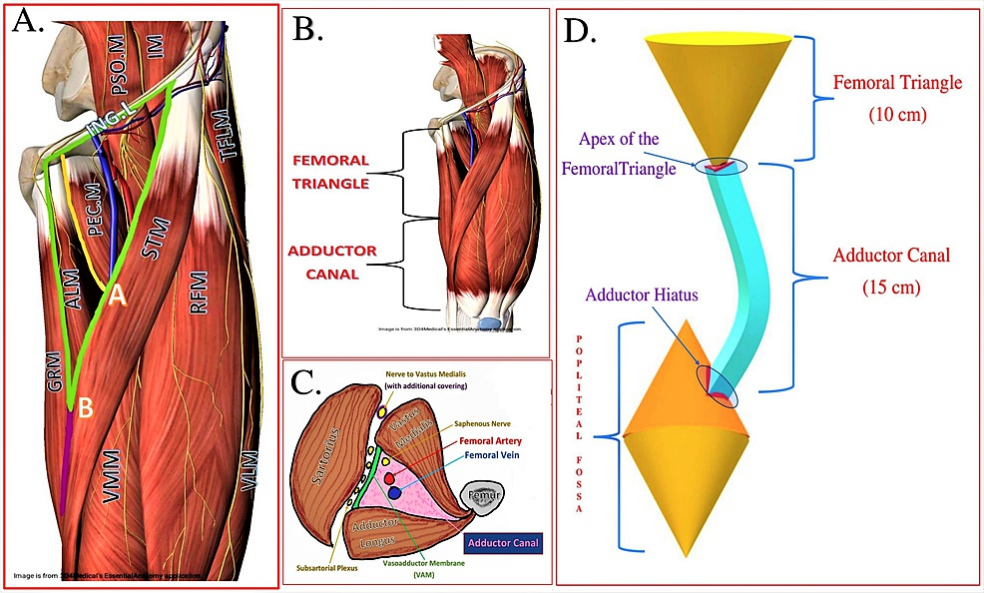
Anatomy of the femoral triangle and the adductor canal (A) Boundaries of the femoral triangle [4]: green lines indicate the border of the femoral triangle; yellow lines indicate the lateral border of ALM; point A indicates the apex of iliopectineal fossa, and point B indicates the apex of the femoral triangle. (B) The extent of the femoral triangle and the adductor canal [4]. (C) Boundaries of the adductor canal [4]. (D) Schematic representation of the adductor canal as a passageway between the anterior thigh to the posterior thigh. STM: Sartorius muscle, RFM: Rectus femoris muscle, TFLM: Tensor fascia lata muscle, VLM: Vastus lateralis muscle, VMM: Vastus medialis muscle, ALM: Adductor longus muscle, GRM: Gracilis muscle, PEC.M: Pectineus muscle, PSO.M: Psoas muscle, IM: Iliacus muscle. Source: Images in Panels A and B are courtesy of the 3D4Medical Essential Anatomy App. Panels A-C were taken from Sonawane et al. [4]. Panel D in this figure was created by the first author KS.

The femoral triangle (FT) and adductor canal (AC) are two prominent landmarks in the thigh that are of great interest for the regional anesthesiologist. It is essential to understand the extent of these landmarks and the separating point between them. The FT is a subfascial triangular space lying in the anterior thigh with the base formed by the inguinal ligament, medial and lateral borders by the medial borders of the adductor longus muscle (ALM) and the sartorius muscle (STM), respectively (Figure 3, Panels A and B). The apex of FT is the intersecting point of the medial borders of ALM and STM (point B in Figure 3, Panel A) that continues as AC distally [3,4].

The AC, a musculoaponeurotic tunnel in the middle third of the thigh, serves as a passageway (Figure 3, Panels C and D) for structures moving from the anterosuperior thigh to the posteroinferior thigh or the posterosuperior leg [4,5]. It is about 15 cm long, extending from the apex of the FT above to the adductor hiatus below [4]. The boundaries of the triangular AC are formed anterolaterally by the VMM, posteromedially by the ALM (in the proximal AC) and adductor magnus (AMM) muscle (in the distal AC), and medially by the vasoadductor membrane (VAM).

**Figure 4:**
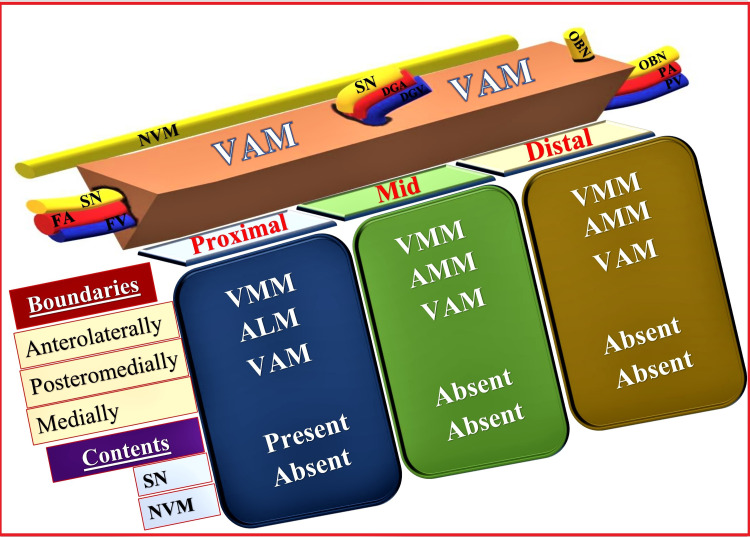
Schematic diagram showing various parts of the adductor canal and their contents SN: Saphenous nerve, FA: Femoral artery, FV: Femoral vein, NVM: Nerve to vastus medialis, VAM: Vasoadductor membrane, DGA: Descending genicular artery, DGV: Descending genicular vein, OBN: Obturator nerve, VMM: Vastus medialis muscle, ALM: Adductor longus muscle, AMM: Adductor magnus muscle. Source: This figure was created by the first author KS.

The VAM is a strong fibrous membrane connecting the anterior and posterior walls of the AC. The STM forms the roof of the AC along with the skin and subcutaneous tissue. The subsartorial plexus occupies the space between the VAM and STM (Figure 3, Panel C). It is formed by SN (infrapatellar branch), obturator nerve (anterior division), nerve to vastus medialis (NVM), and medial femoral cutaneous nerve. The content of the AC includes femoral vessels (femoral artery and vein) and the SN. Femoral vessels exit the AC by passing through an adductor hiatus (a gap between the oblique and the medial head of AMM) and continue as popliteal vessels in the popliteal fossa [6]. The SN in the proximal AC lies lateral to the femoral artery (FA) initially, which later becomes anterior to FA before leaving the AC in the mid-adductor canal area by piercing the VAM along with the descending genicular vessels [2,4] (Figure 4).

Relevant sonoanatomy

**Figure 5:**
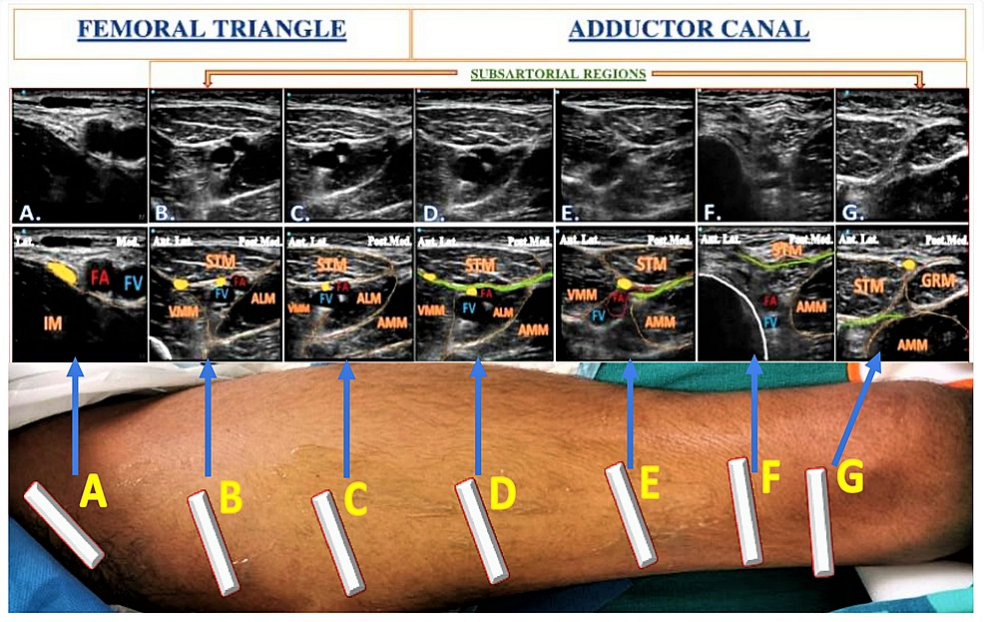
Sonoanatomy at various levels of the thigh (A) Sonoanatomy at the level of the inguinal crease (proximal femoral triangle). (B) Sonoanatomy at the level of the femoral triangle. (C) Sonoanatomy at the level of apex of the femoral triangle. (D) Sonoanatomy at the level of the proximal adductor canal. (E) Sonoanatomy at the level of the mid-adductor canal. (F) Sonoanatomy at the level of the distal adductor canal. (G) Sonoanatomy at the level of distal adductor canal with probe over the medial aspect of the thigh. FA: Femoral artery, FV: Femoral vein, IM: Iliacus muscle, STM: Sartorius muscle, VMM: Vastus medialis muscle, ALM: Adductor longus muscle, AMM: Adductor magnus muscle, Lat.: Lateral, Med.: Medial, Ant.Lat.: Anterolateral, Post.Med.: Posteromedial. Yellow dots indicate the nerves, green lines indicate the vasoadductor membrane, and white lines indicate the bone. Source: Sonawane et al. [4].

The Hi-PAC block is an ultrasound-guided RA technique involving injection into the proximal AC. Therefore, it is essential to find out the apex of FT, a demarcating point between FT and AC under ultrasound. The apex of the FT appears as a figure of “3,” also known as the “kissing sign” [2,4,7] in the ultrasound image, due to overlaying medial borders of ALM and STM. The distal FT is the area just proximal to the apex of FT, while the proximal AC is just distal to the apex of FT. Both the FT and AC regions are also known as subsartorial regions because the STM appears on ultrasound as a common muscular landmark over these regions under ultrasound [2,4,7] (Figure 5).

Three important anatomical events occur in the AC region: (1) entry of femoral vessels and SN through the proximal opening, (2) exit of SN with genicular vessels (branches of femoral vessels) through the anterior opening, and (3) exit of femoral vessels through the distal opening (adductor hiatus). The varied neurovascular inter-relationships resulting from these events divide AC into three parts: proximal, mid, and distal AC [4].

Proximal Adductor Canal

It is the proximal one-third part of AC that roughly occupies a 2-5 cm area distal to the apex of the FT. The sonoanatomy of this region reveals posteromedial ALM, anterolateral VMM, and medial VAM with the STM above (Figure 5, Panel D). The lower border of STM can be seen as bilayered due to the presence of VAM [2,4,7-9]. A hyperechoic SN appears lateral to the FA in the proximal AC. The NVM appears above the VAM in the intermuscular fascial planes between STM and VMM with its additional fascial covering [9-11].

Mid-adductor Canal

It is the middle one-third part of AC lying distal to the proximal AC. The sonoanatomy of this region shows the posteromedial replacement of the ALM by AMM. In this region, the SN with the descending genicular artery (branch of FA) exits the AC by piercing VAM and appears between STM above and VAM below (Figure 5, Panel E), whereas the NVM appears above VAM between STM and VMM. The presence of VAM makes the bilayered appearance of the lower border of STM.

Distal Adductor Canal

It is the distal one-third part of the AC containing femoral vessels but no nerves. At this level, femoral vessels can be seen entering into the adductor hiatus before becoming popliteal vessels. The sonoanatomy of this region reveals posteromedial AMM, anterolateral VMM, and medial VAM with STM above (Figure 5, Panel F).

While scanning from the proximal to the distal part of AC, the SN appears traveling from the lateral-to-anterior side of the FA and becomes superficial by piercing the deep and superficial fascia in the distal-most part of the thigh. The SN crosses AC from the anterior to the medial side and travels from deeper to the superficial plane during its course. Initially, it can be seen between the VMM and STM, then between STM-AMM, and finally between STM and gracilis muscle [2,4,9]. Throughout the AC region, the NVM can be identified as a hyperechoic structure above the VAM between STM and VMM.

Drug distribution in the AC

**Figure 6:**
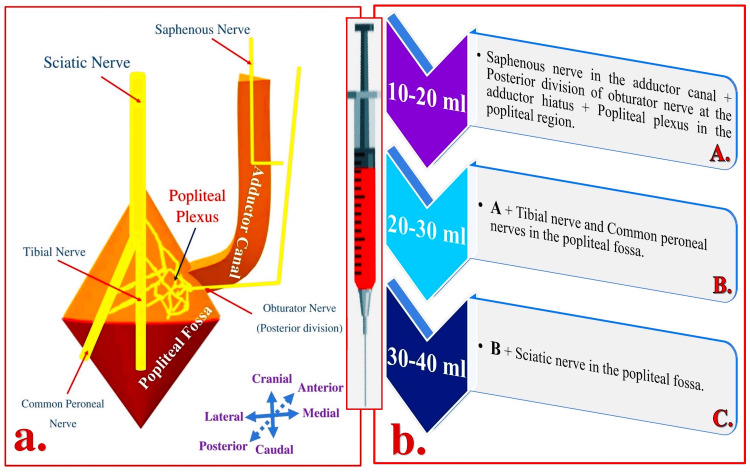
Possible neural components involved in the proximal adductor canal block based on the drug volumes (a) Schematic showing neuroanatomy related to the adductor canal and the popliteal fossa. (b) Possible involvement of the neural components as per the drug volumes in the proximal adductor canal block. Source: This figure was created by the first author KS.

As previously mentioned, the AC is a connecting tunnel or the passageway between the FT anterosuperiorly and the popliteal fossa posteroinferiorly (Figures 3D, 6 [Panel a]). Such a mechanism favors the distal spread of the drug into the popliteal fossa when injected into the AC. However, the distribution of the drug injected into the AC mainly depends on the volume and location of the injection. Femoral vessels entering and exiting the AC serve as a conduit for the injected drug. Thus, a drug injected into the AC below VAM tracks along the femoral vessels and passes through the adductor hiatus where it involves the posterior division of the obturator nerve (which directly enters the adductor hiatus) and then goes to the backside of the knee to include the popliteal plexus [2,4,12,13]. Increasing the injected drug volume in the AC leads to subsequent ascending involvement of the neural components in the popliteal fossa, eventually involving the SCN. The required drug volume in the AC to involve the SCN (in the popliteal fossa) in an adult is 30-40 ml [14].

Technical considerations

Indications and Contraindications

The Hi-PAC block can be an adjunct to the multimodal analgesia regimen to deal with postoperative pain with added motor-sparing advantage for below-knee surgeries. This technique should not be combined with other RA techniques like epidural analgesia, femoral nerve block, or SCN block. Under such conditions, the advantage of the motor-sparing effect of the Hi-PAC block will go in vain.

Choice of Local Anesthetics

The choice of type, volume, and concentration of LA can directly affect the quality of the block. We recommend using lower concentrations and relatively safer LA like 0.1% ropivacaine with an additive such as dexamethasone (8 mg) to improve the quality of the block and the duration of analgesia [15]. The Hi-PAC is a high-volume block requiring 30-40 ml of LA solution to cover all the procedure-specific innervations below the knee joint.

Equipment

Types of equipment during the administration of the Hi-PAC block include:

(1) Ultrasound machine with sterile gel,

(2) High-frequency (6-13 MHz) linear array transducer with a sterile sleeve,

(3) Standard nerve block tray,

(4) One 10-mL syringe containing a local anesthetic solution,

(5) A 100-mm, 21-gauge, short-bevel block needle,

(6) Sterile gloves.

Landmark and Patient Positioning

**Figure 7:**
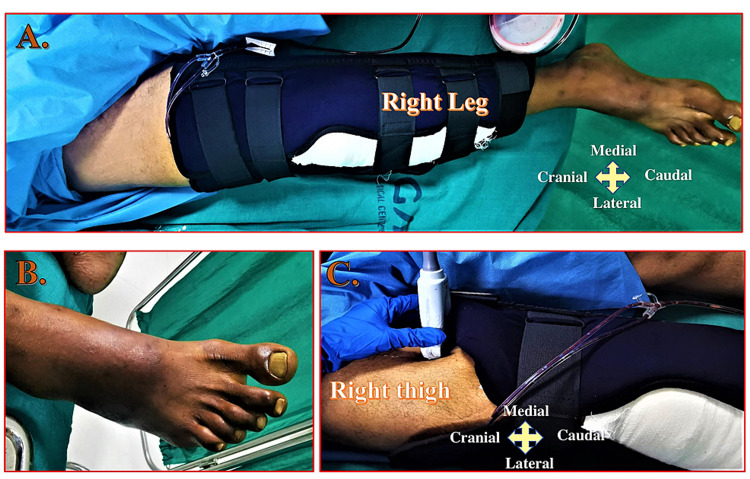
Position of the leg and ultrasound probe for the Hi-PAC block (A) Position of the leg with above-knee brace occupying distal thigh. (B) Looking for toe movements before administrating the Hi-PAC block. (C) Ultrasound probe position over the thigh to locate the proximal adductor canal region. Hi-PAC: High-volume proximal adductor canal.

The Hi-PAC block can be administered by keeping the patient supine with the hip abducted and the thigh externally rotated to facilitate the probe placement and needle insertion. Exposing the entire thigh helps appreciate the distance from the groin to the knee (Figure 7, Panel A).

Timing of Block

The Hi-PAC block provides postoperative analgesia in below-knee surgeries such as the proximal tibia, the shaft of the tibia, ankle, or foot surgeries performed mainly under the neuraxial block. We recommend administering this block only after the patient has begun to move the toes of the affected limbs (Figure 7, Panel B) but before the onset of pain at the surgical site. If general anesthesia (GA) is planned for the surgery, the Hi-PAC block can be administered immediately after induction but before the incision.

Probe Position and Sonoanatomy

The prominent musculatures of the thigh that includes STM, VMM, and ALM can be seen upon placing a high-frequency linear ultrasound probe horizontally over the anteromedial aspect of the thigh (Figure 7, Panel C). The femoral vessels can be identified beneath the STM with the anterolateral VMM and posteromedial ALM. The hyperechoic rim of the femur can be seen under the vastus intermedius muscle.

Upon tracking the ultrasound probe in the craniocaudal direction, the apex of the FT can be identified as overlapping medial borders of the ALM and STM, appearing as a figure of "3." The proximal AC can be located just (2-3 cm) distal to the apex of FT, where a hyperechoic SN can be seen anterolateral to the FA and NVM over the VAM between the VMM and STM. The lower border of the STM can be seen bilayered due to the VAM below it.

Goal

The goal of the Hi-PAC block is to place the linear ultrasound probe in the proximal AC and the needle tip below the VAM immediately adjacent to the FA to target the SN directly and the SCN indirectly through the distal drug spread in the popliteal region.

Steps to Administer Hi-PAC Block

**Figure 8:**
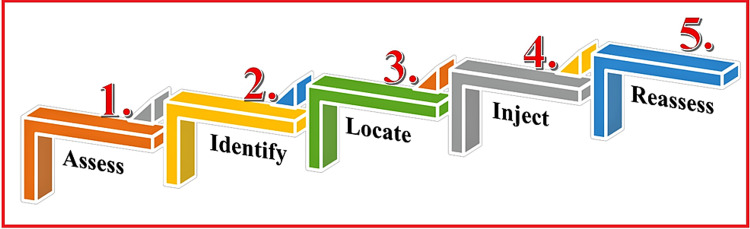
Five steps required to administer the Hi-PAC block Hi-PAC: High-volume proximal adductor canal. Source: This figure was created by the first author KS.

The Hi-PAC block can be administered by five simple steps (Figure 8): (1) Assess the postoperative regression of spinal effect, (2) identify the apex of the FT, (3) locate the proximal AC, (4) inject following the dual-target-single-injection technique, and (5) reassess the motor-sparing effect of the block.

**Figure 9:**
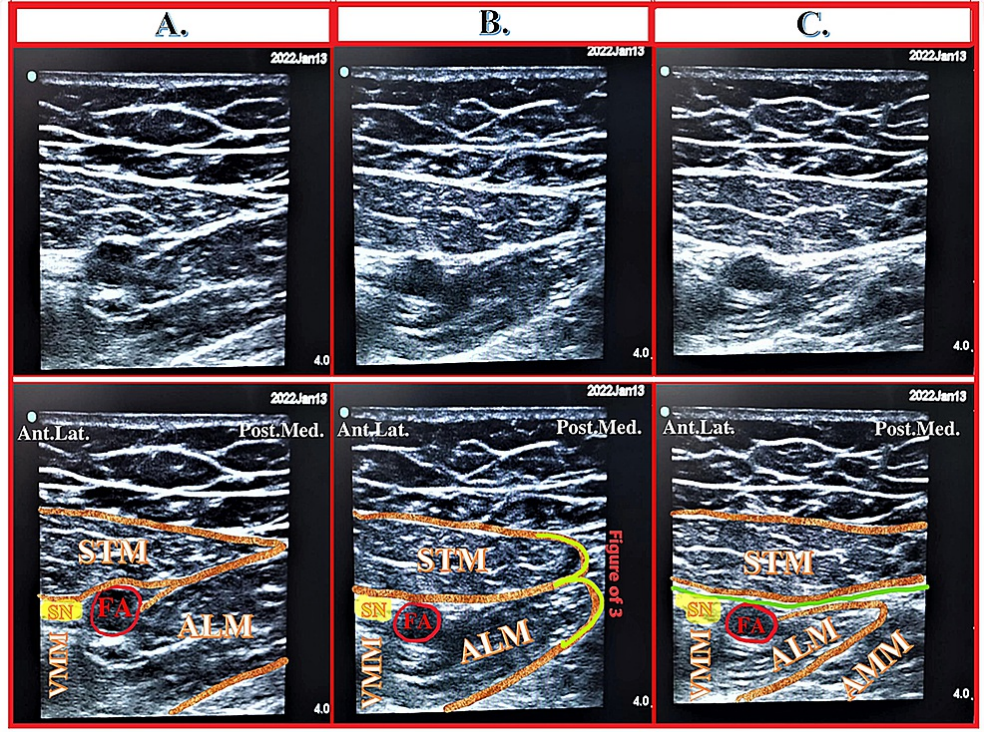
Relevant sonoanatomy of the Hi-PAC block (A) Sonoanatomy at 2-3 cm proximal to the apex of the femoral triangle (distal femoral triangle). (B) Sonoanatomy at the level of the apex of the femoral triangle where medial borders of the sartorius and adductor longus muscles overlie forming a “figure of 3” or “kissing sign.” (C) Sonoanatomy at 2-3 cm distal to the apex of the femoral triangle (proximal adductor canal), which is the target site for Hi-PAC block. Hi-PAC: High-volume proximal adductor canal, FA: Femoral artery; STM: Sartorius muscle, VMM: Vastus medialis muscle, ALM: Adductor longus muscle, AMM: Adductor magnus muscle, SN: Saphenous nerve, Ant.Lat.: Anterolateral, Post.Med.: Posteromedial. The green line below STM indicates the vasoadductor membrane.

**Figure 10:**
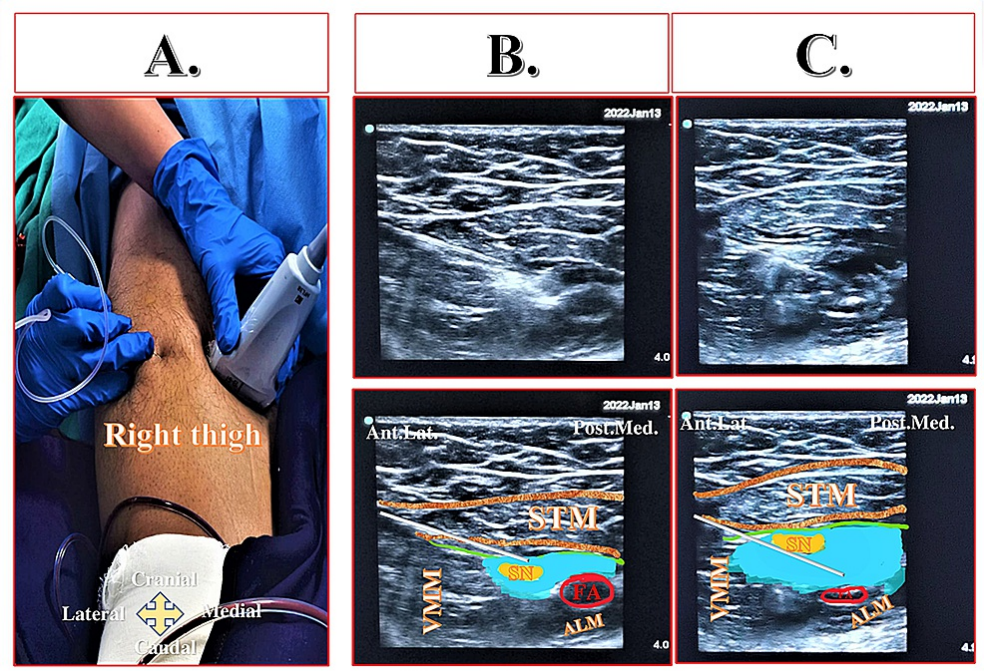
Probe position and dual-target-single-injection technique of the Hi-PAC block (A) Ultrasound probe position and in-plane needle insertion. (B) Deposition of the local anesthetic solution targeting saphenous nerve below the vasoadductor membrane in the proximal adductor canal. (C) Perivascular deposition of the local anesthetic solution below the vasoadductor membrane in the proximal adductor canal. FA: Femoral artery, STM: Sartorius muscle, VMM: Vastus medialis muscle, ALM: Adductor longus muscle, SN: Saphenous nerve, Ant.Lat.: Anterolateral, Post.Med.: Posteromedial, Hi-PAC: High-volume proximal adductor canal. The green line below STM indicates the vasoadductor membrane. The white line indicates the needle. The blue area indicates the drug spread.

1. Assessing the postoperative regression of spinal effect: The Hi-PAC block can be administered only after regaining the toe movements but before the onset of the pain over the surgical area. The presence of a postoperative slab or cast (above or below the knee) makes assessing the other joint movements (knee or ankle) difficult. The motor effect of the spinal anesthesia regains in the distal-to-proximal direction, which can be confirmed solely by observing the toe movements that can be carried out even in the presence of cast/slab.

2. Identifying the apex of the FT: Upon placing the high-frequency linear ultrasound probe over the mid-thigh, muscular landmarks such as anterolateral VMM, posteromedial ALM, and medial STM can be seen. The apex of the FT can be identified as a sign of "3" or "kissing sign," as shown in Figure 9, Panel B.

3. Locating proximal adductor canal: The proximal AC can be located just (2-3 cm) distal to the apex of the FT upon moving the ultrasound probe. After optimizing the ultrasound image, the hyperechoic SN can be identified lateral to the FA (Figure 9, Panel C). Also, bilayering of the lower border of STM due to the presence of VAM can be appreciated.

4. Dual-target-single-injection technique: A 100-mm block needle can be inserted in-plane from the lateral-to-medial direction (Figure 10, Panels A and B) in the plane between STM and VMM. A 10 ml of LA solution can be deposited targeting the SN below the VAM (Figure 10, Panel B). The remaining 30-40 ml of LA solution can be deposited perivascularly adjacent to the FA under the VAM, typically observing compression of the FA upon injecting the drug and regaining its original shape upon stopping the injection (Figure 10, Panel C). We recommend performing this block in the proximal AC, which can be easily accessible even in the presence of slab/cast. Also, it helps to target SN at a fixed location (lateral to FA) and avoid proximity to the sterile surgical dressing area requiring undressing the surgical wound. Due to the proximity of femoral vessels, deposition of LA should involve a slow, incremental injection (5 mL every 10-15 s) with frequent aspiration for blood to avoid intravascular injections.

5. Reassessing the motor-sparing effect of the block: After successful and satisfactory administration of the Hi-PAC block, the motor-sparing effect of the block can be reconfirmed by re-evaluating the active toe movement 30 minutes after the block.

Distribution of Analgesia

**Figure 11:**
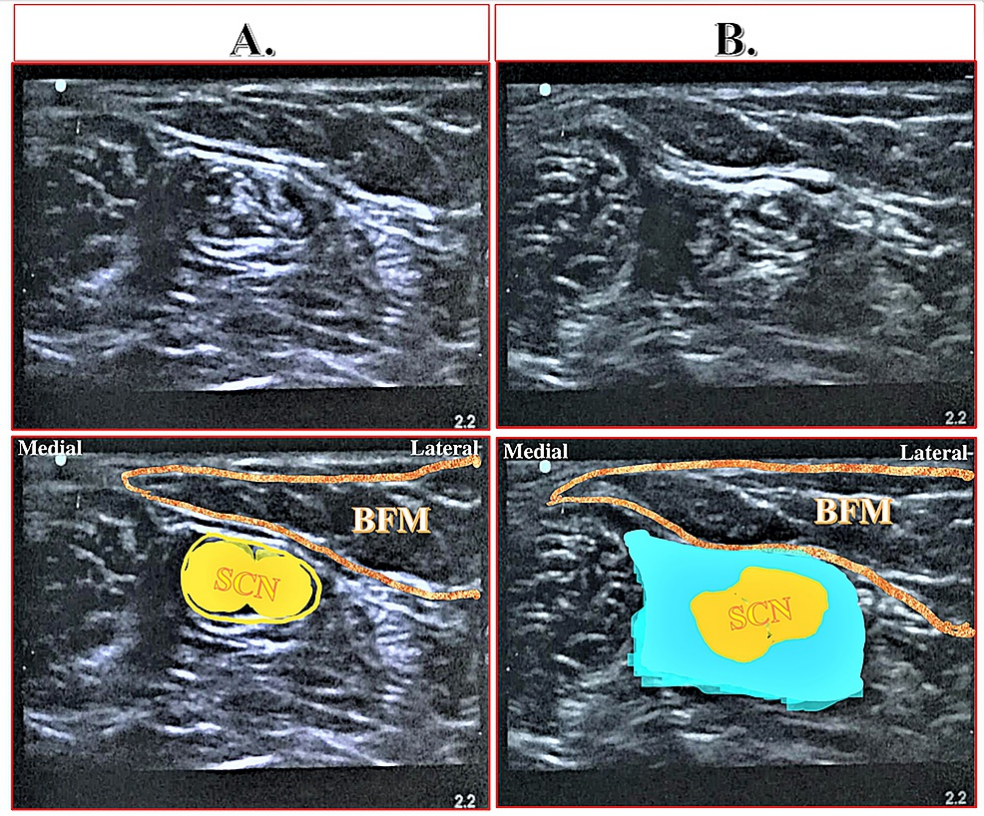
Sonoanatomy showing the sciatic nerve at the level of the popliteal fossa before and after the Hi-PAC block (A) Sciatic nerve before the Hi-PAC block. (B) Sciatic nerve after the Hi-PAC block showing deposition of local anesthetic solution around the nerve due to distal drug spread. The blue area indicates the drug spread. SCN: Sciatic nerve, BFM: Bicep femoris muscle, Hi-PAC: High-volume proximal adductor canal.

In the proximal AC, depositing a high volume (30-40 ml) of LA perivascularly (around FA) under the VAM is sufficient to achieve desired outcomes. This volume and concentration of LA (30-40 ml of 0.1% ropivacaine) are sufficient to block the selective sensory fibers of the SN directly and the SCN indirectly through the distal drug spread into the popliteal fossa. The order of the involvement of the neural components is as follows: SN, the posterior division of the obturator nerve (it enters directly in the adductor hiatus), popliteal plexus (formed by the articular branches from the tibial, common peroneal, sciatic, and posterior division of the obturator nerves), tibial and common peroneal nerve, and the SCN in the popliteal fossa. Indirect drug spread around the SCN can be well appreciated by comparing the pre-block and post-block scanning images of the SCN as shown in Figure 11.

The drug injected into the proximal AC below the VAM tends to spread distally than proximally due to resistance offered by the femoral vessels in the superior opening of the adductor canal. Due to limited proximal spread, the Hi-PAC block may involve NVM in the distal FT but may not involve FN in the proximal FT.

Thus, the proximal and distal drug distribution can result in a selective sensory blockade in the territories of the involved neural components after the Hi-PAC block. These territories may represent the anteromedial aspect of the knee joint (due to NVM and SN block), the medial side of the skin in the leg up to the base of the great toe and the medial malleolus (due to SN block), the intra-articular component of the knee joint and the posterior capsule (due to popliteal plexus block), and the entire leg and foot (due to SCN block).

## Discussion

The Hi-PAC block is a modified AC block administered with several modifications to achieve desired outcomes. These modifications include the fixed location of the block (proximal AC), a novel indication (for below-knee surgery) of AC block (with modifications), fixed timing (postoperative) and prerequisites (after active toe movements) of the block, fixed volume/concentration/type of LA (30-40 ml of 0.1% ropivacaine), dual-target (saphenous nerve and perivascular) single-injection technique, and indirect involvement of SCN due to distal drug spread. These essential modifications make the Hi-PAC block a unique and novel RA technique for below-knee surgeries.

The Hi-PAC block may not cause motor blockade when administered precisely at the exact site and with the LA concentration and volumes as recommended. It can also be considered as one of the subsartorial blocks like several other RA techniques administered in the subsartorial region, including the conventional AC block, distal AC block, and 4-in-1 block (Table 1). The unique qualities of the Hi-PAC block compared to other subsartorial blocks include the feasibility of administration even with the above-knee dressing/slab/cast/brace, relatively easy identification landmarks, and complete coverage of the innervation below-knee joint by direct SN blockade and indirect popliteal SCN blockade through a single anterior injection.

**Table 1:** Comparison of the Hi-PAC block with other described subsartorial blocks ACB: Adductor canal block, Hi-PAC: High-volume proximal adductor canal, HF: High frequency, FT: Femoral triangle, AC: Adductor canal, FA: Femoral artery, SN: Saphenous nerve, NVM: Nerve to vastus medialis, OBN: Obturator nerve, TN: Tibial nerve, CPN: Common peroneal nerve, SCN: Sciatic nerve, LA: Local anesthetics, VAM: Vasoadductor membrane.

Comparison parameters	ACB (conventional)	Distal ACB (as described)	4-in-1 (as described)	Hi-PAC block
Ultrasound probe	Linear HF	Linear HF	Linear HF	Linear HF
Timing of block	Preop/postop	Preop/postop	Preop/postop	Postop only
Location	Mid-thigh (actually corresponds to distal FT region)	Distal thigh (in distal AC)	Distal thigh (in distal AC)	2-3 cm distal to the apex of FT (in proximal AC)
Recommended LA type/concentration	0.5%-0.2% ropivacaine or 0.5%-0.25% bupivacaine	0.5%-0.2% ropivacaine or 0.5%-0.25% bupivacaine	0.2% ropivacaine	0.1% ropivacaine
LA volume	15-20 mL	15-20 mL	30-40 mL	30-40 mL
Drug deposition target	SN lateral to the FA	Above the FA below sartorius muscle	Above the FA below sartorius muscle	SN and FA (perivascular)
Indications	Knee surgeries	Knee surgeries	Knee and below-knee surgeries	Below-knee surgeries
Covered innervations	SN ± NVM	Posterior division of OBN + popliteal plexus	Posterior division of OBN + popliteal plexus + TN and CPN + SCN due to distal spread of LA	SN directly; NVM due to proximal spread; posterior division of OBN + popliteal plexus + TN and CPN + SCN due to distal spread of LA
Analgesia coverage	Anteromedial aspect of the knee joint	Posterior knee, intra-articular knee joint component	Posterior knee, intra-articular knee joint component, SN territory, and the area below knee joint including leg, ankle, and foot	Posterior knee, intra-articular knee joint component, SN territory, and the area below knee joint including leg, ankle, and foot
Possible sparing	± NVM (depends on spread in FT), popliteal plexus, TN, CPN, and SCN	NVM (as it lies above VAM) and SN (as it leaves AC)	NVM (as it lies above VAM) and SN (as it leaves AC)	± NVM (depends on the proximal spread)

This dual-target single-injection technique may be a viable alternative to other RA techniques required to provide postoperative analgesia in below-knee surgeries (Table 2). The Hi-PAC block may be suitable in all below-knee surgeries with the risk of ACS due to its inclusion of diluted LA that selectively targets sensory fibers. It can avoid the delay in diagnosis and treatment of ACS while providing complete postoperative analgesia without masking the symptoms of the impending compartment syndrome. However, periodic postoperative monitoring by trained clinical personnel to look for warning signs and symptoms of ACS in all such surgical procedures is essential.

**Table 2:** Comparison of the Hi-PAC block with other regional analgesia techniques for below-knee surgeries SN: Saphenous nerve, EA: Epidural analgesia, FNB: Femoral nerve block, SCNB: Sciatic nerve block, ACB: Adductor canal block, CAPS: Crosswise approach to the popliteal sciatic nerve, Hi-PAC: High-volume proximal adductor canal, LA: Local anesthetics, ERAS: Enhanced recovery after surgery, AK: Above-knee, Conc.: Concentration.

Comparison parameters	EA	FNB + SCNB (high approaches)	ACB + SCNB (popliteal approach)	CAPS block + SNB	4-in-1 block	Hi-PAC block
Analgesia coverage in the SN distribution	Yes	Yes	Yes	Yes	No	Yes
Analgesia coverage in the SCN distribution	Yes	Yes	Yes	Yes	Yes	Yes
Motor-sparing effect	No	With only low-conc. LA	With only low-conc. LA	With only low-conc. LA	Yes	Yes
Procedure-specific	No	No	Yes	Yes	No	Yes
Opioid-sparing effect	Yes/No	Yes	Yes	Yes	Yes/No	Yes
ERAS suitability	No	No	Yes	Yes	Yes	Yes
Specific patient positioning required?	Yes	Yes	Yes	No	No	No
Number of injections	Single	Double	Double	Double	Single	Single
Suitable to AK casts/slabs	Yes	Yes	No	No	No	Yes
Can mask the symptoms of compartment syndrome?	Yes/No (depends on the conc. of LA)	Yes/No (depends on the conc. of LA)	Yes/No (depends on the conc. of LA)	Yes/No (depends on the conc. of LA)	No	No

Easily identifiable sonoanatomical landmarks and simple injection targets make it easy to teach the Hi-PAC block to RA trainees. However, as with any RA technique, all safety guidelines must be followed to avoid potential complications (Table 3).

**Table 3:** Potential complications of the Hi-PAC block and preventive measures LA: Local anesthetics, LAST: Local anesthetic systemic toxicity, AC: Adductor canal, Hi-PAC: High-volume proximal adductor canal, SN: Saphenous nerve, VAM: Vasoadductor membrane.

Complications	Precautions to avoid complications
Infection	Use strict aseptic techniques and precautions. Clean hands, sterile gloves, probe, needle, and surface.
Surgical site infection	Avoid undressing the surgical wound and proximity to the surgical site by administrating the block in the proximal AC region.
Hematoma	Avoid multiple needle passes. Avoid using needles with a larger diameter.
Vascular injury	It can be avoided by visualizing the entire needle/tip under ultrasound during the entire procedure. Every change in needle directions or depth should be followed by frequent aspirations before injecting LA solutions.
Nerve injury	Slow needle advancement. Avoid injecting drugs at high opening pressures. Use injection pressure monitor if possible.
LAST	Follow frequent aspiration during LA injection and incremental doses of LA while giving the injection. Use LA solution with maximum safety profile such as ropivacaine or levobupivacaine. All standard monitors should be connected to the patient while giving a block.
Myotoxicity	Avoid high-concentration LA solution. Avoid intramuscular drug deposition.
Motor blockade	Avoid giving a higher concentration or volumes of LA than recommended. Avoid additional blockades with Hi-PAC involving the same targets. Recommended Hi-PAC block volume is 30-40 mL in the proximal AC (10 mL around SN + 20-30 mL around FA below VAM).

## Conclusions

The Hi-PAC block can be considered a motor-sparing RA option to provide complete analgesia for below-knee surgeries based on the anatomical and technical considerations. Selective targeting of sensory nerve fibers by using LA at low concentration may fail to mask the pain of impending compartment syndrome, making the Hi-PAC block an appropriate RA technique in such conditions. However, postoperative monitoring and surveillance to identify clinical signs of ACS at set frequencies by trained clinical staff should be continued as a routine protocol in below-knee surgeries at the risk of ACS.

The choice of the Hi-PAC block depends on its comparison with other available RA techniques for below-knee surgeries and the risk/benefits assessment after consultation with the surgical team. It is a novel RA technique that requires a high volume of LA to achieve the desired results, subject to variable analgesic outcomes due to varied or inconsistent drug distribution patterns. However, further large sample-sized clinical, radiographic, or cadaveric dye studies are required to determine the actual drug distribution pattern and exact volume of LA to achieve the desired outcomes of this novel technique.
